# Supporting long‐term engagement in HIV clinical care: Learning from the COVID‐19 pandemic

**DOI:** 10.1111/hiv.70181

**Published:** 2025-12-31

**Authors:** R. Dhairyawan, Sara Paparini, M. Smuk, S. Sidat, R. Mbewe, Silvia Petretti, V. Martin, S. Dakshina, H. Alexander, T. D. Appleby, K. Childs, M. Bracchi, B. Dragovic, G. Haidari, N. Mackie, I. Reeves, A. Umaipalan, L. J. Waters, J. Anderson, C. M. Orkin

**Affiliations:** ^1^ SHARE Collaborative, Blizard Institute Queen Mary University of London London UK; ^2^ Barts Health NHS Trust London UK; ^3^ SHARE Collaborative, Wolfson Institute of Population Health Queen Mary University of London London UK; ^4^ Positively UK London UK; ^5^ Blood Safety, Hepatitis, Sexually Transmitted Infections and HIV Division UK Health Security Agency London UK; ^6^ North Middlesex University Hospital London UK; ^7^ Lewisham and Greenwich NHS Trust London UK; ^8^ King's College Hospital NHS Foundation Trust London UK; ^9^ Chelsea and Westminster Hospital NHS Foundation Trust London UK; ^10^ St George's University Hospital NHS Foundation Trust London UK; ^11^ Guy's and St Thomas' NHS Foundation Trust London UK; ^12^ Imperial College Healthcare NHS Trust London UK; ^13^ Homerton Healthcare NHS Trust London UK; ^14^ Barking, Havering and Redbridge University Hospitals NHS Trust London UK; ^15^ Central North West London NHS Trust London UK; ^16^ Institute for Global Health University College London London UK

**Keywords:** cascade, COVID‐19, engagement, HIV, retention

## Abstract

**Objectives:**

SHIELD is a London‐based study of engagement in HIV clinical care during and after the COVID‐19 pandemic. We document the characteristics of service users who either re‐engaged or disengaged with HIV care, together with reflections of HIV clinical service‐providers to inform recommendations that address care retention for the long term.

**Methods:**

Using an exploratory mixed methods approach, service‐users aged ≥18 years who re‐engaged (1 March 2020–31 August 2020) or disengaged from clinical care (no contact with service 1 March 2020–28 February 2021) at 8 London HIV services were identified by retrospective records review. Demographics and clinical data were summarized descriptively. For people who re‐engaged, follow‐up data were collected at 24 months and in those who disengaged, at a single timepoint (1 March 2023). Semi‐structured interviews with clinic staff were analysed thematically.

**Results:**

There were 122 people disengaged and 89 re‐engaged. In those who re‐engaged, a 24‐month follow‐up showed 71.3% on antiretrovirals, 63.6% virally suppressed and 67.9% had a future clinician appointment booked. For people who disengaged, by 1 March 2023, 21% were on antiretrovirals and 27% had a future appointment booked. Interviews with 11 service providers explored COVID‐19's impact on clinical services, multilevel interactions that influenced engagement, and approaches to re‐engaging people.

**Conclusions:**

We found that COVID‐19 exposed existing vulnerabilities deterring access to HIV clinical care, as well as being itself an additional factor. For some people, the pandemic provided an opportunity to re‐engage. We recommend that retention in care is prioritized in policy and financially, that clinical services provide holistic person‐centred care, and improve ways to identify those who leave care.

## INTRODUCTION

For optimal health outcomes for people living with HIV, lifelong engagement in HIV care and adherence to antiretroviral therapy (ART) are vital and can be challenging to sustain. If the UNAIDS targets of ending AIDS as a public health threat by 2030 are to be met, supporting people to stay on in care is essential. In the UK, the National Health Service (NHS) provides free HIV treatment to everyone living with diagnosed HIV, and the British HIV Association (BHIVA) recommends person‐centred care [1]. However, the United Kingdom Health Security Agency (UKHSA) estimates that in 2023 in England, 4960 (5.3%) of people with diagnosed HIV were not retained in HIV care and 15 800–18 900 (15%–18%) of the 104 000 adults living with HIV had transmissible virus [2]. Re‐engagement is a focus of the new England HIV Action Plan (2025) [3]. Definitions of retention, engagement and disengagement in HIV care are often not explicit in the literature and there is a lack of consensus globally [4]. The UKHSA defines ‘non‐retention in care’ as a person who has not attended (remotely or in person) a specialist clinic within 15 months of their last recorded attendance.

The 2020 COVID‐19 pandemic and related public health measures had a profound impact on HIV services globally, resulting in reduced testing, alterations in clinical encounters and follow‐up, access to ART and medication adherence [5, 6, 7, 8, 9]. In the UK, much HIV clinical care moved from in‐person to remote encounters, and ART supplies were maintained [10]. However, the UKHSA recorded an increase in people not retained in HIV clinical care during the pandemic compared to previous years in England—4732 (5.5%) in 2019, 6670 (7.5%) in 2020 and 6608 (7.4%) in 2021, and down to 4974 (5.5%) in 2022 [2]. They concluded this was a likely consequence of lockdown measures on HIV care access and delivery [2].

Early in the pandemic, staff at Barts Health NHS Trust observed that people who had not been accessing HIV care regularly were contacting clinic. Together with staff at four other NHS HIV clinics in London, data were prospectively collected. Sixty people with documented intermittent or no engagement, were recorded to have re‐engaged with HIV care between 9 March 2020 and 17 May 2020. It was suggested this might reflect a perceived increased risk of COVID‐19 associated mortality and morbidity [11]. To investigate this observation, the *S*ecuring best health outcomes for people living with *HI*V through optimal *e*ngagement with care: *L*earning from *D*isruptive change (the SHIELD Study) was designed. This exploratory study aims to investigate engagement and disengagement in relation to COVID‐19. The study objectives are (1) to characterize service‐users who either re‐engaged or disengaged with HIV care during the pandemic; (2) to review clinical outcomes and engagement in care at 24 months follow up; (3) to explore the views of service‐providers involved in supporting people to remain in care; and (4) to understand the perspectives of service‐users on care engagement. In this paper, we present quantitative data and qualitative service‐provider interviews that relate to the first three objectives.

## MATERIALS AND METHODS

SHIELD is a mixed‐methods study based in London. A quantitative workstream identified and followed people living with HIV who either re‐engaged or disengaged during the COVID‐19 pandemic. Two qualitative semi‐structured interview‐based workstreams explored the views of service providers and users (user‐related findings will be reported elsewhere). A consort diagram detailing workstreams and participating sites is included in the Supporting information.

Eleven study sites were selected from the 27 NHS specialist clinical HIV services located in major NHS hospital Trusts across London. Site selection was purposive to broadly reflect the profile of the 38 680 people (a majority being gay and bisexual men who have sex with men, Black African heterosexual men and women) estimated to be accessing clinical care for HIV in London [12]. Site selection criteria included geographical location (inner or outer London), clinical cohort size (from <500 to >4000 service‐users) and the demographics (deprivation, ethnicity, migration status, sexual orientation) of service‐users. To be selected, sites were required to demonstrate staff capacity to participate and contribute data. The study was co‐designed and implemented with researchers with HIV lived experience and in consultation with the SHARE Collaborative Community Advisory Board.

Definitions of engagement and disengagement can differ between clinicians and patients. Definitions used in this study, detailed in the inclusion criteria of the quantitative workstream, differ from the UKHSA definition of ‘non‐retention in care’, which was introduced after the completion of data collection for this study. Pre‐pandemic, no standardized policies existed across clinical sites to identify and recall patients who did not attend appointments, each having individual procedures.

Ethical approval was granted by the London‐Chelsea Research Ethics Committee (22/LO/0486).

### Service‐user quantitative workstream

People who re‐engaged or disengaged during the COVID‐19 pandemic were identified retrospectively by staff in eight HIV clinical services in London, by systematically searching electronic medical records.

Inclusion criteria

- Aged ≥18 years with a definitive HIV diagnosis
- People who re‐engaged: had not attended a clinic appointment or missed at least 2 appointments between 1 March 2019 and 28 February 2020, and who were in contact with services between 1 March 2020 and 31 August 2020 (by email, telephone or in person).
- People who disengaged: Had attended appointments and were in contact with services before 29 February 2020, but with no contact (telephone, email or in person) with services between 1 March 2020 and 28 February 2021. This was the early pandemic phase which was characterized by repeated lockdowns.

Clinical records of eligible participants were reviewed by local research teams for demographics, HIV clinical and treatment history, and co‐morbidities. In re‐engaged individuals, follow‐up data on attendance, ART use, HIV viral load (VL) and CD4 count were collected 24 months after re‐engagement. Data on ART prescriptions and future appointment booking were collected for the disengaged group at a single timepoint (1 March 2023). Data were collected on a password‐protected Excel spreadsheet and pseudoanonymized at each site before being emailed to the study team for collation, cleansing and analysis. Table 1 shows the variables collected. The criteria for each variable are included in the data collection tool in the Supporting information.

**TABLE 1:** Variables collected.

	People who re‐engaged		People who disengaged
Baseline demographics	Age when re‐engaged	Baseline demographics	Age when disengaged
	Gender identity		Gender identity
	Sexual orientation		Sexual orientation
	Ethnic group		Ethnic group
	Index of Multiple Deprivation decile		Index of Multiple Deprivation decile
	Migration status		Migration status
	Time since diagnosis		Time since diagnosis
	Number of co‐morbidities		Number of co‐morbidities
	Number of mental health diagnoses		Number of mental health diagnoses
	Accessing mental health support		Accessing mental health support
	Smoking		Smoking
	Alcohol		Alcohol
	Recreational drugs		Recreational drugs
	Accessing drug/alcohol support		Accessing drug/alcohol support
	Accessing peer support		Accessing peer support
Baseline clinical data	Date last contacted clinic (remote or in person) Last CD4 count (cells/mm^3^) Last HIV viral load (copies/μL)	Last contact before disengagement	Months since last contacted clinic (remote or in person) Last CD4 count (cells/mm^3^)
	Previously taken ART? (prescription and reduction in viral load)		Last HIV viral load (copies/μL) Was on ART? (prescription and reduction in viral load)
At re‐engagement	Date of re‐engagement Months since last clinic contact or in person	Between disengagement and 1 March 2023	Attended a clinic appointment (remote or in person with any member of HIV team)
	Method of re‐engagement		
	Attended clinician appointment		
	ART script provided?		
	CD4 count (cells/mm^3^)		
	HIV VL (copies/μL)		
Collected at 3, 6, 9, 12, 18, 24 months after re‐engagement	Attended a clinician appointment (remote or in person) Current ART script CD4 count (cells/mm^3^) HIV viral load (copies/μL)	As of 1 March 2023	Future appointment booked Current ART script (indicating individual should have medication)

Abbreviations: ART, antiretroviral therapy; VL, viral load.

Data were analysed using STATA 17. Descriptive statistics were generated, delineating the sample size for each question. For continuous data questions, either the mean with standard deviation or the median with interquartile range was produced to accurately represent the distribution of the data. Categorical data are presented as frequencies and their respective percentages.

### Qualitative interviews with service‐providers

Between 1 November 2022 and 31 May 2023, individual semi‐structured interviews were undertaken with staff from 11 HIV clinical services. Eligible clinical staff were selected by the clinical lead at the site and sampled based on their engagement role in the clinic in supporting people to re‐engage.

A written participant study information sheet and consent form were provided at least one week prior to interview with time to ask questions. Interview topics included: existing service‐level policies supporting engagement in HIV clinical care; changes made during COVID‐19; views on disengagement and how it could be addressed.

The interview topic guide (see Supporting information) was devised by the study team composed of specialist HIV clinicians, qualitative researchers with experience of HIV research and/or lived experience of HIV. Topics were reviewed and refined by the team following two pilot interviews. RD—a British Asian, female, HIV specialist doctor with qualitative and quantitative research experience—conducted all interviews. RD collaborated with SP, a senior qualitative researcher. Interviews were conducted and recorded online, lasted 30–45 min, and were transcribed by an external agency. RD and SP analysed the transcripts using a reflexive thematic approach [13, 14]. The first three interviews were open‐coded independently, producing a coding framework. Following the selection of joint codes, RD and SP coded the remaining data, adding new codes where needed and developed core themes. Themes were presented to, and refined in consultation with, the broader study team and the SHARE Collaborative Community Advisory Board.

## RESULTS

Eight sites took part in the quantitative workstream and eleven in the service‐provider interviews (see consort diagram in the Supporting information).

### Service‐user quantitative workstream

This exploratory analysis includes 122 individuals who disengaged from HIV care, and 89 who re‐engaged during the COVID‐19 pandemic. Descriptive summaries are presented to highlight patterns across demographic, clinical and behavioural factors (Table 2).

**TABLE 2:** Demographic characteristics and clinical outcomes for people who disengaged and people who re‐engaged during the COVID‐19 pandemic.

	Disengaged during COVID‐19 pandemic *N* = 122	Re‐engaged during COVID‐19 pandemic *N* = 89
Gender identity, *n* (%)	*n* = 122	*n* = 89
Cisgender man	86 (70.5)	41 (46.1)
Cisgender woman	35 (28.7)	47 (52.8)
Transgender man	0 (0.0)	1 (1.1)
Transgender woman	1 (0.8)	0 (0.0)
Age (years)	*n* = 117	*n* = 89
Mean (standard deviation)	44.0 (11.4)	43.2 (11.6)
Sexual orientation, *n* (%)	*n* = 120	*n* = 89
Heterosexual men and women	68 (56.7)	66 (74.2)
Gay and bisexual men who have sex with men	52 (43.3)	22 (24.7)
Women who have sex with women	0 (0.0)	1 (1.1)
Ethnic group, *n* (%)	*n* = 115	*n* = 87
Asian	6 (5.2)	2 (2.3)
Black African	31 (27.0)	43 (49.4)
Black British or other	10 (8.7)	11 (12.6)
Black Caribbean	7 (6.1)	3 (3.4)
Mixed	6 (5.2)	5 (5.7)
Other	5 (4.3)	4 (4.6)
White other	37 (32.2)	9 (10.3)
White British	13 (11.3)	10 (11.5)
Index of multiple deprivation deciles^a^, *n* (%)	*n* = 119	*n* = 85
Most deprived (1, 2, 3)	58 (48.7)	47 (55.3)
Intermediate deprivation (4, 5, 6, 7)	50 (42.0)	33 (38.8)
Least deprived (8, 9, 10)	11 (9.2)	5 (5.9)
Migration status, *n* (%)	*n* = 73	*n* = 67
British Citizen	42 (57.5)	33 (49.3)
Limited leave to remain	1 (1.4)	2 (3.0)
Indefinite leave to remain	28 (38.4)	31 (46.3)
Insecure immigration status	1 (1.4)	1 (1.5)
Visitor visa	1 (1.4)	0 (0.0)
Number of co‐morbidities,^b^ *n* (%)	*n* = 122	*n* = 89
0	53 (43.4)	33 (37.1)
1	35 (28.7)	35 (39.3)
2	19 (15.6)	7 (7.9)
3+	15 (12.3)	14 (15.7)
Number of mental health diagnoses,^b^ *n* (%)	*n* = 122	*n* = 89
0	84 (68.9)	65 (73.0)
1	29 (23.8)	14 (15.7)
2	7 (5.7)	7 (7.9)
3	2 (1.6)	3 (3.4)
Accessing mental health support, *n* (%)	*n* = 99	*n* = 72
No	83 (83.8)	55 (76.4)
Yes	16 (16.2)	17 (23.6)
Smoking, *n* (%)	*n* = 104	*n* = 80
Current smoker	25 (24.0)	25 (31.3)
Ex‐smoker	20 (19.2)	11 (13.8)
Never smoked	41 (39.4)	42 (52.5)
Not currently smoking	18 (17.3)	2 (2.5)
Alcohol, *n* (%)	*n* = 103	*n* = 78
Drinks excessively	6 (5.8)	7 (9.0)
Drinks in moderation	54 (52.4)	36 (46.2)
Not drinking	43 (41.7)	35 (44.9)
Recreational drugs, *n* (%)	*n* = 117	*n* = 89
Currently using	28 (23.9)	17 (19.1)
Not using	89 (76.1)	72 (80.9)
Accessing drug/alcohol support, *n* (%)	*n* = 112	*n* = 82
Yes	7 (6.3)	9 (11.0)
No	105 (93.8)	73 (89.0)
Accessing peer support, *n* (%)	*n* = 108	*n* = 84
Yes	3 (2.8)	8 (9.5)
No	105 (97.2)	76 (90.5)
Last CD4 count prior to disengagement^c^	*n* = 120	*n* = 76
Median cells/mm^3^ (Interquartile range)	562.0 (385.5–744.0)	442 (272.0–690.0)
Was on ART prior to disengagement, *n* (%)	*n* = 118	*n* = 88
Yes	98 (83.1)	82 (93.2)
No	20 (16.9)	6 (6.8)
Undetectable HIV viral load (<200 copies/μL) on ART prior^c^ to disengagement, *n* (%)	*n* = 97	*n* = 75
Yes	84 (86.6)	47 (62.7)
No	13 (13.4)	28 (37.3)

^a^ Index of multiple deprivation (IMD) deciles rank small geographic areas from 1 (most deprived 10% of areas) to 10 (least deprived 10% of areas).

^b^ Numbers of co‐morbidities and mental health diagnoses: Counts reflect the number of comorbidities and mental health diagnoses listed by participants in free‐text responses. Entries were reviewed and categorized by experts before being tallied.

^c^ For participants classified as disengaged, the CD4 count and HIV viral load shown are the last recorded values prior to 29 February 2020; the interval between these measurements and disengagement varies by individual. For participants classified as re‐engaged, the measurements are prior to 1 March 2019 and represent their last recording before disengagement. This varies by individual.

Gender identity differed between groups: most disengaged people identified as cisgender men (70.5%), whereas cisgender women made up the majority of those re‐engaged (52.8%). Both groups were similar in age (mean ~44 years). In terms of sexual orientation, gay and bisexual men who have sex with men were more common among the disengaged group (43.3%), while heterosexual people were more prevalent among those re‐engaged (74.2%). Ethnic distribution varied: Black African people represented nearly half of the re‐engaged group (49.4%), while White Other backgrounds were more common among those disengaged (32.2%). Most people came from areas of high deprivation, particularly among the re‐engaged group (55.3% in deciles 1–3). Among those with migration data, British citizenship and indefinite leave to remain were the most common statuses in both groups. Over 40% of both groups reported no co‐morbidities, though a slightly higher proportion of re‐engaged people reported three or more. Mental health conditions were more prevalent in the disengaged group, while more re‐engaged people were accessing mental health support. There were small differences in substance use; more re‐engaged individuals were current smokers, and slightly more were accessing drug, alcohol, or peer support services. Clinically, a higher proportion of re‐engaged individuals had been on ART prior to disengagement (93.2% vs. 83.1%), but viral suppression was more common in the disengaged group (86.6% vs. 62.7%). Median CD4 counts before disengagement were also higher in the disengaged group.

Overall, people who disengaged tended to be more clinically stable (e.g., higher CD4 count, viral suppression) and less connected to support services, while individuals who re‐engaged appeared to face greater structural or health‐related challenges but were more often accessing formal support.

#### People who re‐engaged

First contact with their clinic was mostly initiated by patients with a phone call 48.3%, walk‐in 21.3%, or email 4.5% (Table 3).

**TABLE 3:** Methods of re‐engagement.

Method, *n* (%)	*n* = 89
Phone call	43 (48.3)
Email	4 (4.5)
Walk in	19 (21.3)
Inpatient admission	6 (6.7)
Referral	1 (1.1)
Other	16 (18)

At re‐engagement, the median CD4 count was 347.0 cells/mm^3^ (IQR 147.5–597.0) and 32.5% had a VL <200 copies/mL. At 24‐month follow‐up, the median CD4 count remained 347.0 (IQR 216.0–585.0), 71.3% had a current ART prescription and 63.6% had a VL <200 copies/mL. Two‐thirds of this group had a future clinician appointment booked (67.9%) after the 24‐month follow‐up.

#### People who disengaged

At 1/3/23, 33% had attended a clinic appointment with any member of the HIV clinic team (in person or remotely), 21% had a current ART prescription, and 27% had a future appointment booked (Table 4).

**TABLE 4:** Status at 1 March 2023 for people who disengaged.

Attended a clinic appointment, *n* (%)	*n* = 122
Yes	40 (32.8)
No	82 (67.2)
Future appointment booked, *n* (%)	*n* = 122
Yes	33 (27.0)
No	89 (73.0)
Current ART script, *n* (%)	*n* = 122
Yes	25 (20.5)
No	97 (79.5)

### Qualitative interviews with service‐providers

A total of 11 participants from 11 London HIV clinics took part in interviews: 6 doctors, 2 specialist nurses, 2 health advisors and 1 clinic manager. Quotes are included in Table 5.

**TABLE 5:** Findings from semi‐structured interviews with service‐providers.

Theme	Representative quote
Impact of COVID‐19 on HIV clinical services	‘*COVID gave us the confidence to stay in touch with people in different ways and do telephone appointments and attend anywhere. And I think that's made a huge improvement*’.
	‘*Post COVID there's a sense I think that some people are forced to experience care locally, and have realized actually that it's very, very good. And that fear they've had for many years. is about accessing local services, thankfully disappeared*’.
	‘*In 2020 basically, we sent everyone a six‐month supply of medication, regardless of anything, didn't look at any blood test results. And hoped for the best*’.
Harmful factors to engagement in care	‘*I think it's one thing getting people tested, but the hard thing is keeping them going. We're expecting to see them for the rest of their lives. I think that's been quite underestimated*’.
	‘*Very difficult lives, poverty, housing, family problems, immigration issues. So, they have a hefty load of that, combined with some level of stigma. And I think the combination of those, the stigma plus the competing need is quite deadly, because they don't really want to think about the HIV anyway, and they've got other things that they are worried about more, especially if they feel well*’.
Protective factors to engagement in care	‘*I think it's key to have someone in clinic all the time, who is really able to devote time. I could easily spend my whole week doing this work*’.
	‘*We have a housing and benefits advisor who comes into service once a week. We have an alcohol drugs worker who comes into the service once a week. We have a masseur reflexologist, who does a couple of sessions a week. We've got a peer support gardening group*’.
	‘*I really feel it's things being a bit more individual and a bit more personal. That works. People really respond to somebody that they know or know of*’.
Means of re‐engagement	‘*It's just quite protocolised, you try and get hold of the patient by every means that you've got. You email. You text them, you call them, you try them at different times of day. You try them from numbers they may not recognize because they won't necessarily pick up if they realize that [hospital] is calling them where we've got consent. You liaise with the GP…You put we put alerts on their A&E records, particularly if we've got reason to think they're immunosuppressed*’.
	‘*We also say to people, you are welcome to walk in at any time, and I am around in the background and fairly flexible, And that is something we try to say on those patient records don't ever turn, you know, don't turn that patient away. They must be seen*’.

#### Impact of COVID‐19 on HIV clinical services

COVID‐19 was a step into the unknown, often accelerating previously considered changes to clinic policies, including less frequent monitoring, longer prescription durations, home delivery, and remote appointments. The pandemic required service‐providers to review communication with patients, for example, the introduction of a mobile telephone for service‐users to contact. Service‐providers felt that some of these adaptations would be useful to continue post‐pandemic. However, COVID‐19 reinforced inequalities such as digital exclusion (a lack of access to the internet and other digital technologies), which could become further barriers. Participants described how some out‐of‐London service users decided to register with their local clinics, despite previous resistance.

#### Harmful factors to engagement in care

Staff discussed factors they regarded as harmful to engagement based on their professional experience. At person level, participants described how social and economic factors, treatment fatigue, competing priorities, stigma and shame, low self‐esteem and past trauma negatively affect engagement. They noted these factors were synergistic. Service providers described a sense of responsibility, acknowledging that the work of supporting engagement was often relational and serious and could be an additional burden on their workload. Many acknowledged the limits to what they could do and that much effort was needed for what was sometimes a perceived low likelihood of achieving re‐engagement or retention. Some felt these activities were under‐prioritized and under‐resourced at health‐system level with precarious funding for linked support services, a lack of consistent definitions of engagement, reliable data systems and data sharing across clinics, with resources prioritized for diagnostic testing instead.

#### Protective factors to engagement in care

Participants discussed service‐level factors they regarded as protective to patient engagement. These included accessibility, flexibility, continuity of care, non‐judgemental, personalized and holistic approaches, all being considered pivotal to building trust and long‐lasting patient‐centred relationships. They emphasized the importance of always welcoming service‐users back, with a willingness to provide a safe space and ensure the clinic is built around the needs of the service‐users. Processes were created to identify struggling patients, including provision of mental health support and linking to peer and non‐clinical support services. However, in‐clinic peer‐support workers were not always available, which was considered a barrier to access and uptake of peer support.

#### Means of re‐engagement

The commitment to re‐engaging patients was palpable. Although some provider‐specific processes had been created, no service had standardized methods to predict those at risk of disengaging. Processes included IT solutions such as computerized alerts for missed appointments, relying on re‐diagnosis via opt‐out testing in emergency departments, a one‐stop multidisciplinary clinic and several forms of communication media. Offers which went beyond the medical model, including reflexology, housing and alcohol services, were seen as helpful. Service‐providers described the use of ‘any means necessary’ to contact the patient, from a dedicated phone to home visits to involving other care providers. Individuals were usually discussed in multidisciplinary teams as a form of intensive case support.

## DISCUSSION

In London, we found the COVID‐19 pandemic led to ongoing disengagement of some individuals and to successful re‐engagement of others who had high rates of virological suppression two years later. We describe the demographics of both groups. For those who disengaged, few returned despite efforts from the clinics. Service‐provider interviews contextualized our quantitative findings, exploring the impact of COVID‐19 on services, protective and harmful factors for engagement in care and means of re‐engagement (during COVID‐19 and more generally).

Studies pre‐pandemic found disengagement from care associated with younger age, deprivation, being racially minoritized, poor mental health and substance/alcohol misuse [15, 16, 17, 18]. National data from 2019 to 2022 showed younger people (15–34 years) and people born abroad were less likely to be in care compared to older people and those born in England [6]. Our study (excluding people aged <18 years) showed that for re‐engaged and disengaged groups, median age was in the early 40s and half lived in the three most deprived IMD deciles. High levels of mental illness and substance/alcohol misuse were noted within both groups. We found differences by ethnic group, gender and sexual orientation between the groups. A large proportion of people who disengaged were from the White Other ethnic group who may have relocated to their countries of origin during the pandemic; a limitation was that country of birth was not collected and we do not know if people did leave London. An initiative in South London in 2020–2021 found that the people who were re‐engaged were more likely to be women and/or of Black ethnicity, similar to our findings where a large proportion of people who re‐engaged were cis‐women and of Black African ethnicity [19].

Our results show that people who disengaged during the pandemic were either stable on treatment with high CD4 counts and suppressed HIV VL, or had never taken ART. Service providers mentioned treatment fatigue and this may be one reason why ‘stable patients’ disengaged. Many of the people in the re‐engaged group had lower CD4 counts and higher VLs before they disengaged, suggesting they had already experienced engagement challenges. A third (32.5%) of the group that re‐engaged (29/89) had a HIV VL of <200 copies/mL suggesting people had adequate supplies of ART, possibly stored, or were seen elsewhere. These findings demonstrate that predicting who may disengage from HIV care is not always easy, and this may include patients deemed to be ‘stable’.

This unpredictability may be expected. It has been proposed that engagement in HIV care is a ‘revolving door’ rather than a linear continuum—a cycle with periods of engagement and disengagement due to vulnerabilities or other major life stressors [20]. Many of the service users from both groups in our study spent time in and out of HIV care before, during and after the pandemic. However, no service providers had clinic policies to anticipate who may be at risk of disengaging from care. Instead, procedures were reactive, identifying people already out of care.

Service‐providers described pandemic adaptations, including switching from in‐person to telephone appointments, reducing monitoring blood tests, lengthening ART prescriptions and delivering medication home. A national survey of HIV clinicians in the first COVID‐19 wave showed such changes replicated in the rest of England [6]. Some service‐providers felt that digital innovations were still useful post‐pandemic. However, there was concern about digital exclusion, especially for those living in poverty, which was also highlighted in global studies [21, 22, 23, 24, 25, 26]. A survey of people living with HIV in England found that 92% had at least one remote consultation during COVID‐19, but while 73.6% had private access to the internet, this was lower for women, Black African people and those aged ≥65 years [27].

Service‐providers emphasized the considerable resources needed to support people with engagement. The lack of data systems within clinics and co‐ordination between services described is concerning and suggests that services may not always be aware of people not in care. This may partially explain why two‐thirds of the disengaged group were not back in care two years later by March 2023. Service providers also offered a range of definitions for ‘dis/engagement’ as commonly reported globally [4]. Staff described how COVID‐19 exacerbated poor mental health, social and economic factors which increase vulnerability to disengagement, also reported in global studies [5, 28, 29, 30, 31].

Encouragingly, at 24‐month follow up, 63.6% of the re‐engaged group had a suppressed HIV VL and service‐providers reported clinic strategies to re‐engage and retain people in care. Most were similar to interventions seen globally, including intensive case support, a holistic approach, enhanced data monitoring, flexibility in accessing clinics, peer support, lived experience navigators, psychosocial support, help with transport costs and a variety of ways to communicate with patients [15, 19, 21, 22, 23]. An Integrated Care Board‐funded initiative in South London with a dedicated team successfully re‐engaged 75% of people not in care over 12 months, showing that when resource is provided, retention in care can be markedly improved [19]. Few individuals in our study had accessed peer support, suggesting barriers to this essential service remain. Providers noted that patients were more likely to turn the offer down if peer support was not provided in‐house.

A study strength was that it was co‐designed and implemented with people with lived experience of HIV who contributed to study design, data collection and analysis. Participants were recruited from a diverse range of clinics. A limitation is that this study is London focused and may not reflect the experience of providers elsewhere. As it was a retrospective review based on routinely collected service data, we were unable to describe full participant flow as recommended in STROBE, which limits comparability with studies where such information is available. Other limitations include missing data in the quantitative workstream due to 3 sites not being able to provide data and potentially missing eligible participants, and knowing whether people who are not back in HIV care are truly disengaged. They may, instead, have transferred care abroad, or may have passed on. The quality of the quantitative data may have suffered with several clinics reporting changes to their IT systems since 2020, making access to historical data challenging.

## CONCLUSION

We found that COVID‐19 both unveiled existing vulnerabilities which deterred people from accessing HIV clinical care and was itself a vulnerability for some. For others, it provided an opportunity to re‐engage.

The new England HIV Action Plan encourages retention in care, which we welcome [3]. Our qualitative findings indicate that it is currently under‐resourced so we recommend commissioners specify parity with HIV testing initiatives in both funding and policy focus [31]. This should include sustainable investment in community nursing, dedicated re‐engagement staff in clinics and HIV voluntary sector organizations that deliver social and peer support, in‐house wherever possible. Systematic methods of identifying those not in care and data sharing between services should be implemented. The new UKHSA definition of ‘non‐retention in care’ and forthcoming BHIVA guidance on retention in care should support this. Service‐providers should increase flexibility of access to service‐users, train staff to ensure they are welcoming to people re‐engaging and promote continuity of care.

Our quantitative findings show that service‐provider interventions supported some people to stay in care, while not others. It is essential to understand from service‐users themselves what works and what does not work. Findings from our service‐user qualitative interviews will be published. Global, national and local ambitions to end the HIV pandemic will only be met through lifelong engagement of people living with HIV in clinical care. Our study reinforces the need for supporting engagement in HIV care to be a priority for service providers and policy‐makers.

## AUTHOR CONTRIBUTIONS

The study was conceptualised by RD, JA and CMO. RD and CMO acquired the study funding. The methodology, recruitment plan and dissemination strategy were determined by RD, MS, SP, SS, RM, SPe, JA and CMO. RD, HA, TA, KC, MB, BD, GH, NM, IR, AU and LW were responsible for data collection at their study sites. The curation and formal analysis of the quantitative survey and clinical data was conducted by RD and MS. The qualitative interview data was collected and curated by RD, and the formal qualitative analysis was conducted by RD, RM and SS with supervision from SP. The study findings were interpreted by RD, MS, SP, RM, SS, JA and CMO. The original draft of the manuscript was written by RD, MS, SP, JA and CMO. Reviews and edits of subsequent drafts were conducted by all co‐authors.

## CONFLICT OF INTEREST STATEMENT

RD has received speaker fees from ViiV Healthcare and Gilead Sciences. SP has received research grants from ViiV Healthcare and Gilead Sciences. SD has received support to attend a conference from ViiV Healthcare. NM has received advisory fees from ViiV Healthcare and Gilead Sciences. IR has received speaker and consultancy fees from ViiV Healthcare, Gilead Sciences and MSD. AU has received advisory fees from Gilead Sciences. LJW has received speaker/advisory fees from ViiV Healthcare, MSD, Janssen & AbbVie, and is an investigator on trials sponsored by Gilead Sciences, ViiV Heathcare and MSD. JA has received speaker and consultancy fees from Gilead Sciences and speaker fees from ViiV Healthcare. CMO has received honoraria for advisory boards, lectureships and travel sponsorships from Janssen, Gilead Sciences, ViiV Healthcare, MSD and Bavarian Nordic, and has received research grants from Janssen, Gilead Sciences, ViiV Healthcare, MSD and AstraZeneca. The remaining authors (MS, SS, RM, SP, VM, HA, TDA, KC, MB, BD, GH) have no interests to declare.

## Supporting information


**Data S1:** Consort diagram revision clean.

**Data S2:** Service‐provider interviews topic guide.

**Data S3:** SHIELD Quantative WS data collection tool.

## Data Availability

The data that support the findings of this study are available on request from the corresponding author. The data are not publicly available due to privacy or ethical restrictions.
