# Supplementary material for: Supporting long‐term engagement in HIV clinical care: Learning from the COVID‐19 pandemic
Source: HIV Med. 2025 Dec 31;27(5):678–89. doi: 10.1111/hiv.70181 (PMC13140006; doi:10.1111/hiv.70181)
Supplement: Supplementary file 1 — Data S1: Consort diagram revision clean. [file HIV-27-678-s003.docx]

**SHIELD Study Site Participation Consort Diagram**

Qualitative workstream 1

Service Provider interviews

n= 11 sites

11 participants

Qualitative workstream 2

Service User interviews

n= 4 sites

14 participants

Excluded (n= 3 sites)

- unable to collect data due to changes in electronic patient record systems since 2020.

Excluded (n= 4 sites)

- Unable to recruit service users for interviews

Quantitative workstream

n= 8 sites

Data on 211 individuals collected
