# Supplementary material for: Supporting long‐term engagement in HIV clinical care: Learning from the COVID‐19 pandemic
Source: HIV Med. 2025 Dec 31;27(5):678–89. doi: 10.1111/hiv.70181 (PMC13140006; doi:10.1111/hiv.70181)
Supplement: Supplementary file 2 — Data S2: Service‐provider interviews topic guide. [file HIV-27-678-s001.docx]

**SHIELD Study Workstream 1 (Service providers) Interview Topic Guide**

Introductions and discussing the information sheet and consent form

Check the participant has enough privacy and feels they are able to speak freely.

Please be assured this information is only being collected for the purpose of research and none of it will be identifiable.

Do not switch on the tape recorder until the participant has read and signed the information sheet and consent form and has had a chance to ask any questions.

**A) The HIV service (general)**

- What sort of organisation does your HIV service belong to? (eg foundation trust, community trust)
- Tell me about the geographical area and population your HIV organisation provides for. (size of area, transport links, socioeconomic mix, ethnic diversity, key resources)
- Tell me about the patient cohort that use your service.

(size of cohort, mix of gender, sexuality, age, ethnicity, religion, inclusion groups, socioeconomic status, migrant/citizenship status, vulnerability/complexity).

- What services does your organisation provide for people living with HIV?

(clinical HIV, clinical non-HIV such as mental health/dietician, social, psychological, peer-support, welfare advice, other)

**B) Your role**

- Can you tell me about your role within the service? (job type, how long have you worked there, how long in this current role)
- What are the other roles that make a difference to how you do your role in the clinical service? (ie is there someone in your clinic who helps recall patients, who helps you with your patient admin).

**C) Working with patients who disengage from care.**

**I’m going to ask a set of Qs about your organisation first, and then ask for your own opinion afterwards**

- In your opinion is disengagement from care at your service common or rare?
- How would your service define a patient that is lost to follow up?
- How do you know which patients have disengaged from care?

(is this something monitored, and if so, how?)

- Do people usually disengage and re-engage in your service (*prompt:* do they engage for a while, then dis-engage – is this a cycle?)
- Does the organisation have a way of identifying (formally or informally) someone who might be ‘at risk’ of disengagement?
- Does your organisation have any specific services for patients who find it difficult to engage with services? (in person, virtual, community appointments)
- What does your organisation do (if anything) to try and re-engage people who have dis-engaged into care?
- In practice, in your opinion, how well do the interventions put in place work, to keep patients in care?

**D) Your understanding of why patients disengage from care**

- Why do you (rather than your organisation) think some patients find it difficult to engage in HIV care?
- What do you think helps people stay engaged in your service?
- Are there parts of the service that people tend to engage with more/better?
- Is there more your organisation could be doing to support people to remain engaged in care? What about healthcare organisations in general?
- Do you think other factors outside of the clinic can help people to stay engaged in HIV care (within the community, economically, socially or in any other way)?
- What do you think about the role of peer support from other people living with HIV in supporting people living with HIV stay engaged in care? (This may be from direct experience of peer support offered in the clinic or more generally?)

End of interview

- Ask participant if there is anything else they want to add about the topic.
- Thank participant
- Ask participant if they have any questions
- Thank participant again
